# Modulatory effects of dietary tannins on polyunsaturated fatty acid biohydrogenation in the rumen: A meta-analysis

**DOI:** 10.1016/j.heliyon.2022.e09828

**Published:** 2022-06-29

**Authors:** Malik Makmur, Mardiati Zain, Muhammad Miftakhus Sholikin, Suharlina, Anuraga Jayanegara

**Affiliations:** a IPB University, Bogor, Indonesia; b Department of Animal Nutrition, Faculty of Animal Science, Andalas University, Padang, Indonesia; c National Research and Innovation Agency, Jakarta Pusat, Indonesia; d East Kutai Agricultural College School, Sangatta, Indonesia; e Department of Nutrition and Feed Technology, Faculty of Animal Science, IPB University, Bogor, Indonesia; f Animal Feed and Nutrition Modelling (AFENUE) Research Group, Department of Nutrition and Feed Technology, Faculty of Animal Science, IPB University, Bogor, Indonesia

**Keywords:** Digestion, Linolenic acid, Meta-regression, Polyphenol, Research synthesis

## Abstract

**Background:**

Tannins are a group of phenolic compounds that can modify the rumen biohydrogenation (BH) of polyunsaturated fatty acids (PUFA), but to date results obtained have been inconsistent. This study therefore aims to conduct a meta-analysis of the scientific literature related to the effects of tannins on rumen BH and fermentation.

**Methods:**

A total of 28 articles were collected from various scientific databases, such as Scopus, Science Direct and Google Scholar, and the data were analysed using a random effects model and meta-regression for rumen BH. The publication bias on the main variables of rumen fermentation was assessed using a funnel plot and Egger's test.

**Results:**

An increase in tannin levels significantly reduced methane production (p < 0.001) and the population of *Butyrivibrio fibrisolvens* (p < 0.05). Dietary tannins also decreased the SFA proportion (p < 0.001) and increased (p < 0.001) the rumen monounsaturated fatty acid (MUFA) and polyunsaturated fatty acid (PUFA) proportions. In additions, there were negative relationships between dietary tannin levels and BH rates of C18:2 n-6 and C18:3 n-3 (p < 0.05).

**Conclusion:**

Dietary tannins modulate the rumen fermentation profile, mitigate methane emissions, and inhibit rumen BH of PUFA.

## 1 Introduction

Polyunsaturated fatty acids (PUFA) are part of the essential fatty acids, and therefore need to be supplied through diets rich in the substances. The provision of PUFA at elevated levels in animal products (meat, milk, etc.) has gained attention due to their beneficial effects for human health [1]. As the proportion of PUFA in animal products increases, the SFA content decreases, which causes the PUFA/SFA ratio in meat to increase. According to Poulson et al. [2], the content of C18:2 c9 t11 (rumenic acid) in the longissimus and semitendinosus muscles increases by 200%–400% during the pasture-based finisher period. A previous study demonstrated that the intake of PUFA plays an important role in maintaining human health through its metabolic role as an anticarcinogen [3].

Most of the PUFA consumed by ruminants pass through metabolic processes by rumen microbes from the genus *Butyrivibrio* sp. Accordingly, in the rumen system lipolysis and biohydrogenation (BH) processes convert PUFA to SFA, especially C18:0 (stearic acid) and a small proportion of C18:1 t11 (vaccenic acid). Extensive BH activity causes ineffective deposition of PUFA in animal products. The presence of plant secondary metabolites such as phenols and tannins may affect the lipolysis and BH of PUFA in the rumen. It has been shown that tannins reduce PUFA BH and increase PUFA concentration in the rumen [4, 5, 6, 7, 8], but the results have varied.

A meta-analysis of the effect of dietary tannins on the BH activity of PUFA in the rumen has yet to be conducted. This indicates that further verification of the strategic function of tannins as modulators of rumen lipid metabolism is required. The *in vitro* and *in sacco* studies conducted by Jayanegara et al. [6], Jafari et al. [9] and Jafari et al. [10] showed the potential of various tropical forage species as a tannin source in modulating BH to increase the flow of C18:3 n-3 and C18:2 n-6 to the duodenum. Several studies have also shown that some tropical forage species can improve production performance by providing bypass protein for ruminants [11, 12]. Therefore, the meta-analysis is expected to provide a comprehensive evaluation of the effects of dietary tannins on the fermentation, fatty acid profile and PUFA BH activity in the rumen based on various scientific literature sources.

## 2 Materials and methods

### 2.1 Database development

**Figure 1:**
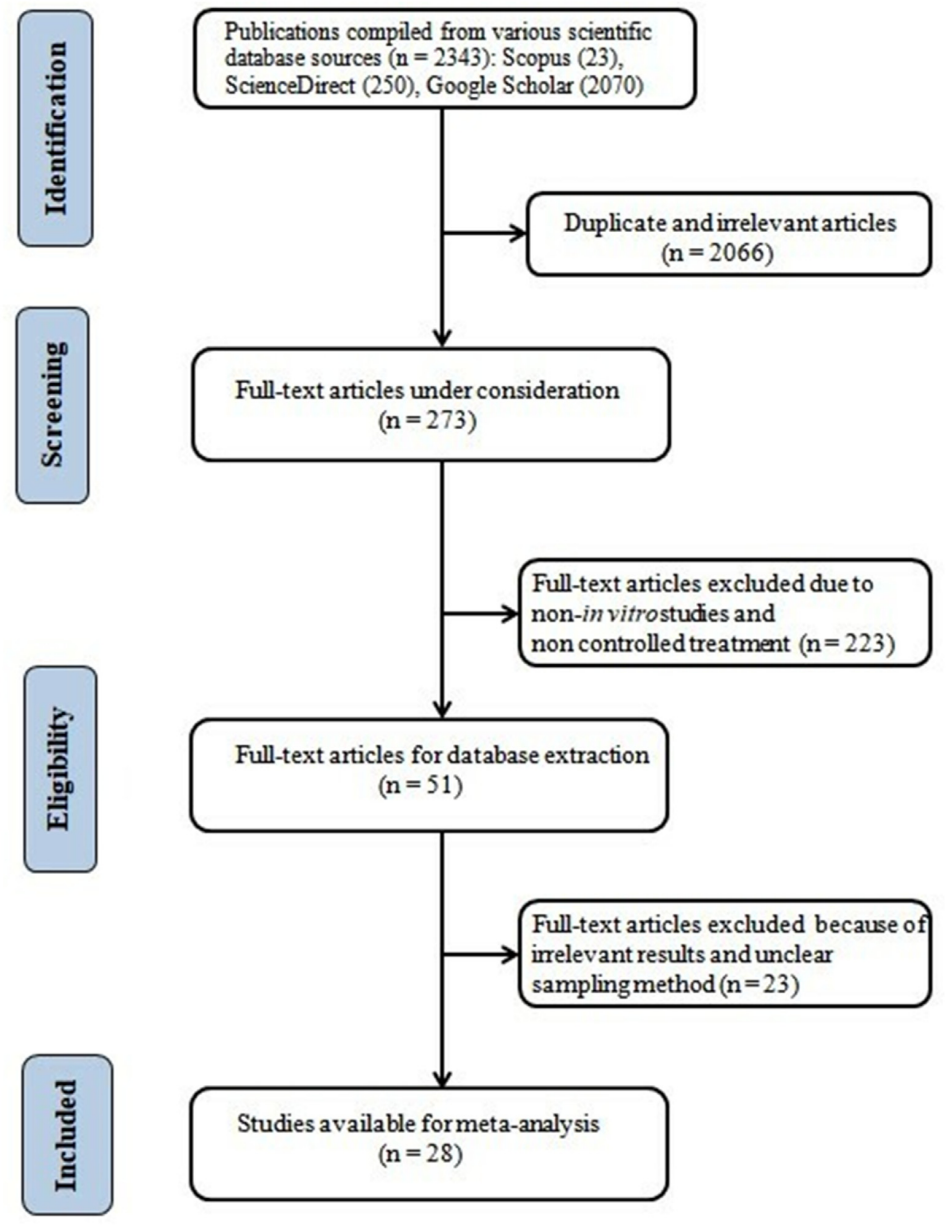
Flow chart of scientific literature search and selection.

A database was developed from studies on the use of dietary tannins on the profile of rumen fermentation, fatty acids, and the BH of PUFA. The scientific literature search engines used were Scopus, Google Scholar, and Science Direct with the keywords “tannin”, “*in vitro*”, “rumen”, “fatty acid”, and/or “biohydrogenation”. The inclusion criteria for articles in the meta-analysis study were: (1) those published in English; (2) inclusion of control treatment in the experiment (no addition of tannin); (3) the presence of tannin sources in the basal diet or as additives; and (4) the experiment was evaluated through the rumen *in vitro* system. The literature search and selection process in the meta-analysis are shown in Figure 1. A total of 51 full-text articles were selected according to the inclusion criteria, but 23 were removed due to irrelevant experimental data and sampling methods that did not meet the criteria set. The number of studies that finally met the criteria was 28 (Table 1), with the process based on the Preferred Reporting Items for Systematic Reviews and Meta-Analysis (PRISMA) protocol [40]. The tabulated *in vitro* rumen fermentation techniques consisted of the Hohenheim gas test (n = 5), the *in vitro* gas production system (n = 6), batch culture incubation (n = 1), the pressure transducer technique (n = 3), glass bottle incubation (n = 11), the rumen simulation technique (n = 2), and the Hungate tube (n = 1). The rumen fluid donors were taken from cows, goats and sheep. Articles were published over the period 2009 to 2022, with a tannin level ranging from 0 to 40.1% DM.

**Table 1:** *In vitro* study included in the meta-analysis database.

No.	Reference	*In vitro* method	Incubation time (h)	Rumen donor	Basal feed	Tannin type	Tannin source	Tannin level (% DM)
1	Abo-Donia et al. [13]	HGT	6, 12, 24	Goat (Liuyang black)	Maize stover and concentrate (45:55)	Hydrolysable tannin	Gallnut	0–0.9
2	Aiman-Zakaria et al. [14]	IGPS	24	Goat (Kacang crossbred)	Alfalfa hay and concentrate (50:50)	Condensed tannin	*Elaeis guineensis* leaf	0–10
3	Al-Jumaili et al. [15]	IGPS	24	Goat (Kacang crossbred)	Alfalfa hay and concentrate (50:50)	Tannic acid	Commercial tannic acid	0–20
4	Bichara [16]	GBI	3, 6, 9, 24	Cow (Holstein)	Grass silage, linseed oil	Condensed tannin	*Inga edulis*, *Desmodium ovalifolium*	0–8.15
5	Buccioni et al. [17]	GBI	6, 12, 18	Sheep (Ewes)	Wheat straw and concentrate	Tannin	Quebracho, chestnut	0–8.2
6	Cappucci et al. [18]	PTT	6,12, 24	Sheep (Massese ewes)	Barley, wheat bran, dehydrated alfalfa and concentrate	Tannin	*Acacia dealbata*, *Uncaria gambir*, *Casealpinia spinosa*, *Castanea sativa*	0–4
7	Carreño et al. [19]	GBI	12, 24	Sheep (Merino)	Alfalfa hay:concentrate (50:50)	Tannin	*Schinopsis lorentzii*, *Vitis vinifera*, *Castanea sativa*, *Quercus robur*, *Quercus petraea*	0–8
8	Costa et al. [20]	GBI	6	Sheep	Dehydrated alfalfa, wheat grain, soybean meal, sunflower oil	Tannin	Chestnut, quebracho, grape seed, rockrose	0–10
9	Fatahnia et al. [21]	GBI	24	Cow (Holstein)	Alfalfa hay:wheat straw (70:30)	Tannic acid	Commercial tannic acid	0–0.07
10	Guerreiro et al. [22]	HT	6	Sheep (Merino Branco)	Dehydrated alfalfa, wheat grain, soybean meal, sunflower oil	Condensed tannin	*Cistus ladanifer*	0–10
11	Guerreiro et al. [23]	GBI	24	Sheep (Merino Branco)	Oat hay and concentrate	Condensed tannin	*Cistus ladanifer*	0–10
12	Irawan et al. [24]	HGT	24, 48	Cattle (Bali)	Forage:concentrate (75:25), corn oil	Hydrolysable tannin	*Leucaena leucocephala*	0–4
13	Ishlak et al. [25]	BCI	24	Cow (Holstein)	Grass hay: concentrate (44:56)	Condensed tannin	Quebracho	0–10
14	Jafari et al. [9]	IGPS	24	Goat (Kacang crossbred)	Alfalfa hay:concentrate (60:40)	Tannin	*Carica papaya* leaf	0–6
15	Jafari et al. [26]	IGPS	24	Goat (Kacang crossbred)	Alfalfa hay:concentrate (50:50)	Condensed tannin	*Carica papaya* leaf	0–6
16	Jafari et al. [27]	IGPS	24	Goat (Kacang crossbred)	Alfalfa hay:concentrate (50:50)	Condensed tannin	*Carica papaya* leaf	0–15
17	Khiaosa-ard et al. [28]	RUSITEC	24	Cow (Brown Swiss)	Grass-clover hay	Condensed tannin	*Acacia mearnsii*, *Onobrychis viciifolia*	0–7.9
18	Mandal et al. [29]	GBI	24	Goat	Barseem hay:concentrate mixture (40:60), sunflower oil	Tannin	*Artocarpus heterophyllus*, *Ficus benghalensis*, *Ficus glomerata*	0–1
19	Menci et al. [30]	PTT	24	Sheep (Texel breed)	TMR, hay:concentrate (80:20)	Condensed tannin, mixture hydrolysable and condensed tannin	*Castanea sativa*, *Schinopsis lorentzii*	0–3
20	Minieri et al. [31]	HGT	6, 12, 18	Sheep (Ewes)	Grass hay and concentrate	Condensed tannin	*Schinopsis lorentzii*	0–4.9
21	Miri et al. [32]	HGT	24	Cow	Hay:concentrate (50:50)	Tannin	*Azadirachta indica*, *Allium sativum*, *Cuminium cyminum*, *Terminalia chebula*	0–40.1
22	Natalello et al. [33]	GBI, PTT	12, 24	Sheep (Merino)	TMR, forage:concentrate (50:50)	Tannin	Whole pomegranate by-product	0–2
23	Odhaib and Sazili [34]	HGT	24	Sheep (Dorper)	Ammoniated rice straw:concentrate (60:40)	Tannin	*Nigella sativa* seeds, *Rosmarinus officinalis* leaves	0–2
24	Shokryzadan et al. [35]	IGPS	24	Goat	Alfalfa:concentrate (60:40)	Condensed tannin	*Garcinia mangostana*	0–8.4
25	Szczechowiak et al. [36]	RUSITEC	24	Cow (Polish Holstein-Friesian)	TMR, maize silage, lucerne silage, concentrate	Condensed tannin	*Vaccinium vitis idaea*	0–0.45
26	Thanh et al. [37]	IGPS	24, 48	Cow (Holstein-Friesian)	Forage:concentrate (60:40), soybean oil	Condensed tannin	Grape seed	0–0.8
27	Toral et al. [38]	GBI	6, 24	Sheep (Ewes)	Alfalfa hay, sainfoin hay	Condensed tannin	*Onobrychis viicifolia*	0–3.5
28	Vasta et al. [39]	GBI	12	Cow (Friesian-Holstein)	Hay, hay plus concentrate	Tannin	*Ceratonia siliqua*, *Acacia cyanophylla*, *Schinopsis lorentzii*	0–0.1

### 2.2 Data analysis

The data were analysed using the random effects meta-analysis method. The effect size calculation (d) in Eq. (1) was based on the standardised mean difference of Hedges' d [41]:

(1) $$d=\frac{\left({\bar{\text{X}}}^{\text{E}}-{\bar{\text{X}}}^{\text{C}}\right)}{S}\;J$$

where ${\bar{\text{X}}}^{\text{E}}$ is the mean of the experimental or tannin group; ${\bar{\text{X}}}^{\text{C}}$ is the control group; *S* the pooled standard deviation; and *J* the correction factor for the small sample size. The mathematical modeling of the one-way random effect is stated in Eq. (2).

(2) $$y_{i}=\;\theta \;+v_{i}+\;\varepsilon_{i}$$

where *y*_*i*_ is the value of the effect size (in Hedge’s d); *θ* the *i*-th observation (the general parameter of the combined effect size; *v*_*i*_ the real variation of the effect size; and ε_i_ the error of the *i*-th observation. In Eq. (3), the estimation of the variance between studies (*τ*^2^) was based on the DerSimonian and Laird [42] method:

(3) $$\tau^{2}=\frac{Q-df}{C}$$

where *Q* is the weighted sum square; *df* the degrees of freedom; and *C* the value of C. The meta-analysis was conducted using the OpenMEE platform (http://www.cebm.brown.edu/openmee/) for rumen fermentation variables (15 items), rumen fatty acids (20 items), and C18 UFA BH (3 items). A cumulative forest plot (95% confidence interval) and meta-regression of the tested variables were constructed using MedCalc software (https://www.medcalc.org/). Subsequently, a funnel plot and Egger's test were employed to detect publication bias both visually and quantitatively, performed using JASP software (https://jasp-stats.org/).

## 3 Results

### 3.1 Rumen fermentation

**Figure 2:**
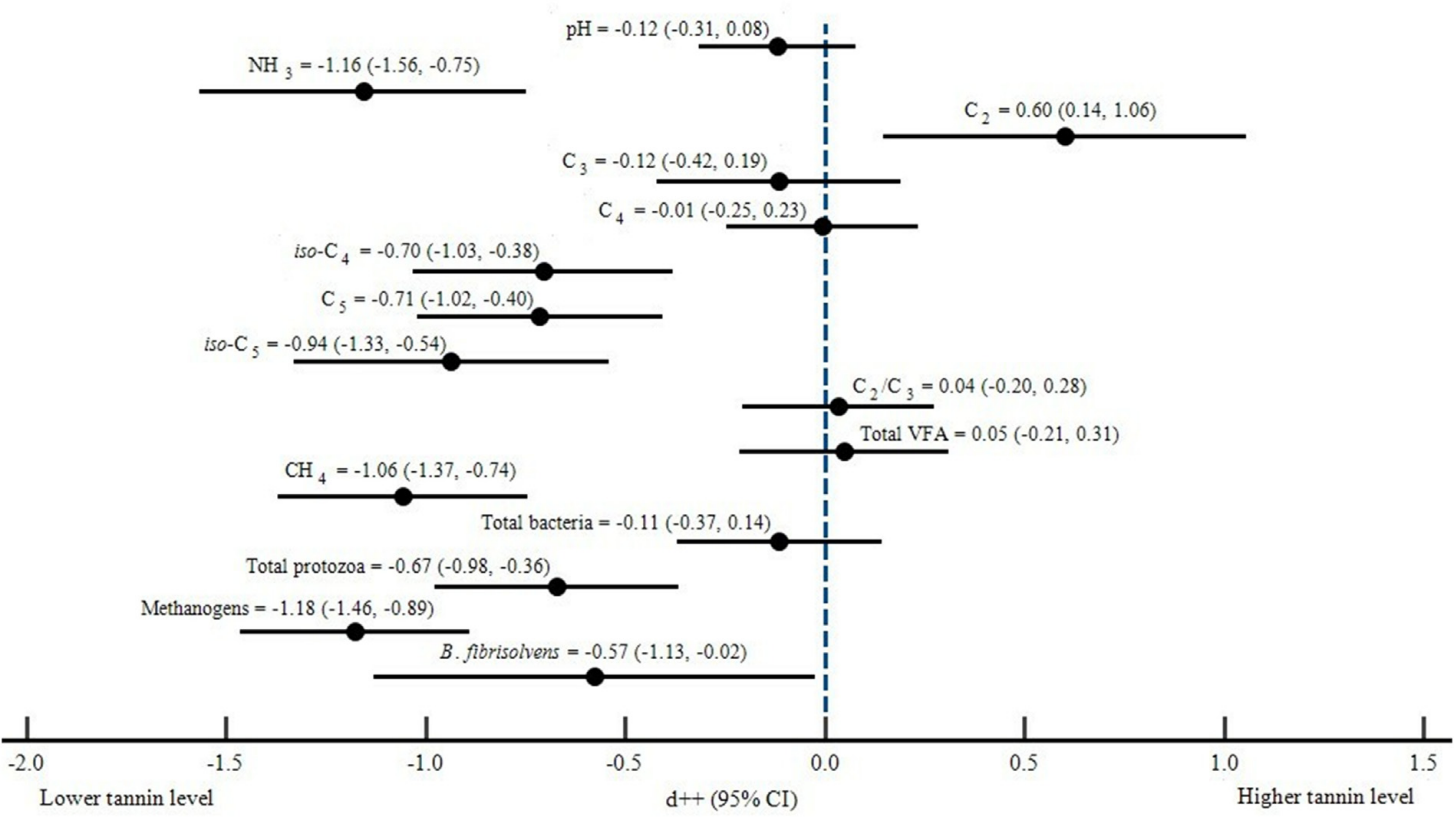
Cumulative forest plot for effect size of the rumen fermentation variable.

Descriptive statistics of various parameters in the database are presented in Table 2. The results of the meta-analysis in Table 3 show that, in comparison to the control, dietary tannins significantly decreased (p < 0.001) the concentrations of ammonia, (NH_3_), valerate (C_5_), *iso*-butyrate (*iso*-C_4_), and *iso*-valerate (*iso*-C_5_). Furthermore, tannins significantly reduced (p < 0.05) the population of protozoa, methanogens, and *B. fibrisolvens* bacteria in the rumen. Methane formation under *in vitro* conditions also decreased significantly (p < 0.001) in the tannin group. However, dietary tannins did not significantly alter pH, several VFA items (total VFA, C_3_, C_4_, and C_2_/C_3_ ratio), and total bacteria. The cumulative forest plot results for each effect size of rumen fermentation profiles are shown in Figure 2.

**Table 2:** Descriptive statistics of the database.

Variables	Unit	NC	Mean		MIN		MAX		SD	
			Control	Tannin	Control	Tannin	Control	Tannin	Control	Tannin
Rumen fermentation										
pH		69	6.86	6.85	6.37	6.38	7.40	7.46	0.27	0.29
NH_3_	mg/dL	66	23.24	19.81	8.40	5.01	70.90	65.40	11.89	11.75
C_2_	mM	64	49.47	50.73	11.91	5.01	83.74	104.48	19.85	22.88
C_3_	mM	64	22.62	23.17	4.37	4.14	33.46	42.4	7.09	7.80
C_4_	mM	64	11.19	11.37	2.07	1.88	23.57	24.94	8.01	7.74
*iso*-C_4_	mM	32	1.43	1.17	0.15	0.01	5.60	4.90	1.75	1.34
C_5_	mM	32	2.50	2.18	0.19	0.15	7.50	6.50	2.33	1.91
*iso*-C_5_	mM	32	2.00	1.75	0.17	0.03	5.45	5.82	2.12	1.94
C_2_/C_3_		64	2.55	2.54	1.61	1.12	3.52	5.03	0.66	0.74
Total VFA	mM	64	67.06	69.43	6.40	6.00	126.36	153.96	34.61	38.89
CH_4_ 24 h	ml/g DM	28	7.55	5.89	3.24	2.99	11.12	8.85	2.27	1.82
Total bacteria	Log _10_cells/L	14	10.21	10.06	8.94	8.75	11.18	11.06	0.78	0.79
Total protozoa	Log _10_cells/L	14	6.38	5.43	5.07	4.79	7.48	6.52	1.00	0.46
Methanogens	Log _10_cells/L	14	8.13	7.38	6.81	5.94	9.06	8.66	0.80	0.79
*B. fibrisolvens*	Log _10_cells/L	11	4.47	3.93	3.37	3.51	5.33	4.60	0.88	0.37
Rumen FA profile										
C14:0	% total FA	16	2.81	2.01	0.62	0.63	5.69	3.02	1.89	0.44
C15:0	% total FA	56	1.03	0.94	0.37	0.23	2.72	2.41	0.64	0.59
C16:0	% total FA	16	16.71	15.72	1.27	1.24	19.62	23.23	6.09	6.33
*iso*-C16:0	% total FA	34	2.36	1.93	0.10	0.09	15.50	16.10	4.89	3.90
C16:1 n-7	% total FA	32	0.97	0.79	0.44	0.23	1.66	1.49	0.39	0.37
C17:0	% total FA	27	4.76	3.88	0.42	0.18	37.00	54.90	11.62	11.21
*iso*-C17:0	% total FA	32	2.30	2.04	0.29	0.36	13.70	21.60	4.38	4.35
C18:0	% total FA	58	33.06	30.37	4.08	2.60	61.90	59.10	18.39	16.36
C18:1 n-9	% total FA	64	7.64	8.99	0.10	0.13	35.80	43.58	8.21	10.01
C18:1 t10	% total FA	16	2.29	2.27	0.36	0.30	4.58	5.57	1.77	1.80
C18:1 t11	% total FA	30	11.89	15.93	4.38	4.31	22.70	43.50	6.13	9.67
C18:2 n-6	% total FA	25	5.28	6.82	2.37	1.20	10.85	19.93	2.89	5.99
C18:2 c9 t11	% total FA	76	0.56	0.60	0.09	0.03	4.38	5.20	1.11	0.93
C18:2 t10 c12	% total FA	60	0.50	0.54	0.01	0.02	4.30	5.10	0.89	0.99
C18:3 n-3	% total FA	70	0.82	1.07	0.15	0.15	3.90	5.98	1.01	1.47
C20:4 n-6	% total FA	25	0.86	0.82	0.01	0.01	2.73	2.26	0.81	0.62
C20:5 n-3	% total FA	21	0.85	1.12	0.04	0.05	1.97	2.80	0.60	0.76
SFA	% total FA	44	57.66	52.80	36.12	29.30	83.06	82.79	14.89	16.21
MUFA	% total FA	36	16.48	19.84	7.56	5.25	26.20	36.76	5.94	9.67
PUFA	% total FA	69	9.48	12.62	3.54	2.71	21.57	34.97	5.85	8.73
C18 UFA biohydrogenation										
C18:1 n-9	%	46	45.90	42.38	17.00	10.22	75.92	72.00	23.71	22.15
C18:2 n-6	%	53	69.10	64.56	31.00	25.10	90.54	89.03	19.83	18.40
C18:3 n-3	%	61	74.46	70.04	25.71	36.30	93.50	90.25	18.14	15.90

**Table 3:** Effect of dietary tannin on the *in vitro* rumen fermentation variable.

Variable	Unit	NC	Estimate	Lower bound	Upper bound	Std. error	*p*-Value	τ^2^	Q	Het. *p*-value	I^2^
pH		69	−0.119	−0.314	0.077	0.100	0.235	0.220	113.335	<0.001	40.001
NH_3_	mg/dL	66	−1.156	−1.566	−0.747	0.209	<0.001	2.087	330.919	<0.001	80.358
C_2_	mM	64	0.601	0.145	1.057	0.233	0.010	2.790	483.613	<0.001	86.973
C_3_	mM	64	−0.116	−0.422	0.190	0.156	0.458	1.246	305.428	<0.001	77.081
C_4_	mM	64	−0.007	−0.247	0.233	0.122	0.953	0.641	194.343	<0.001	62.438
*iso*-C_4_	mM	32	−0.706	−1.031	−0.382	0.166	<0.001	0.613	137.151	<0.001	77.397
C_5_	mM	32	−0.714	−1.022	−0.406	0.157	<0.001	0.630	152.239	<0.001	75.696
*iso*-C_5_	mM	32	−0.936	−1.330	−0.541	0.201	<0.001	0.988	192.446	<0.001	83.892
C_2_/C_3_		64	0.035	−0.205	0.275	0.122	0.774	1.058	519.834	<0.001	81.340
Total VFA	mM	64	0.049	−0.213	0.311	0.134	0.715	0.776	252.441	<0.001	75.044
CH_4_ 24 h	ml/g DM	28	−1.058	−1.370	−0.745	0.160	<0.001	0.354	56.116	<0.001	51.885
Total bacteria	Log_10_ cells/L	14	−0.114	−0.370	0.142	0.131	0.384	0.000	12.115	0.518	0.000
Total protozoa	Log_10_ cells/L	14	−0.672	−0.979	−0.366	0.156	<0.001	0.091	17.729	0.168	26.675
Methanogens	Log_10_ cells/L	14	−1.178	−1.466	−0.890	0.147	<0.001	0.033	14.577	0.335	10.816
*B*. *fibrisolvens*	Log_10_ cells/L	11	−0.576	−1.130	−0.023	0.282	0.041	0.589	32.125	<0.001	68.872

### 3.2 Rumen fatty acids

**Figure 3:**
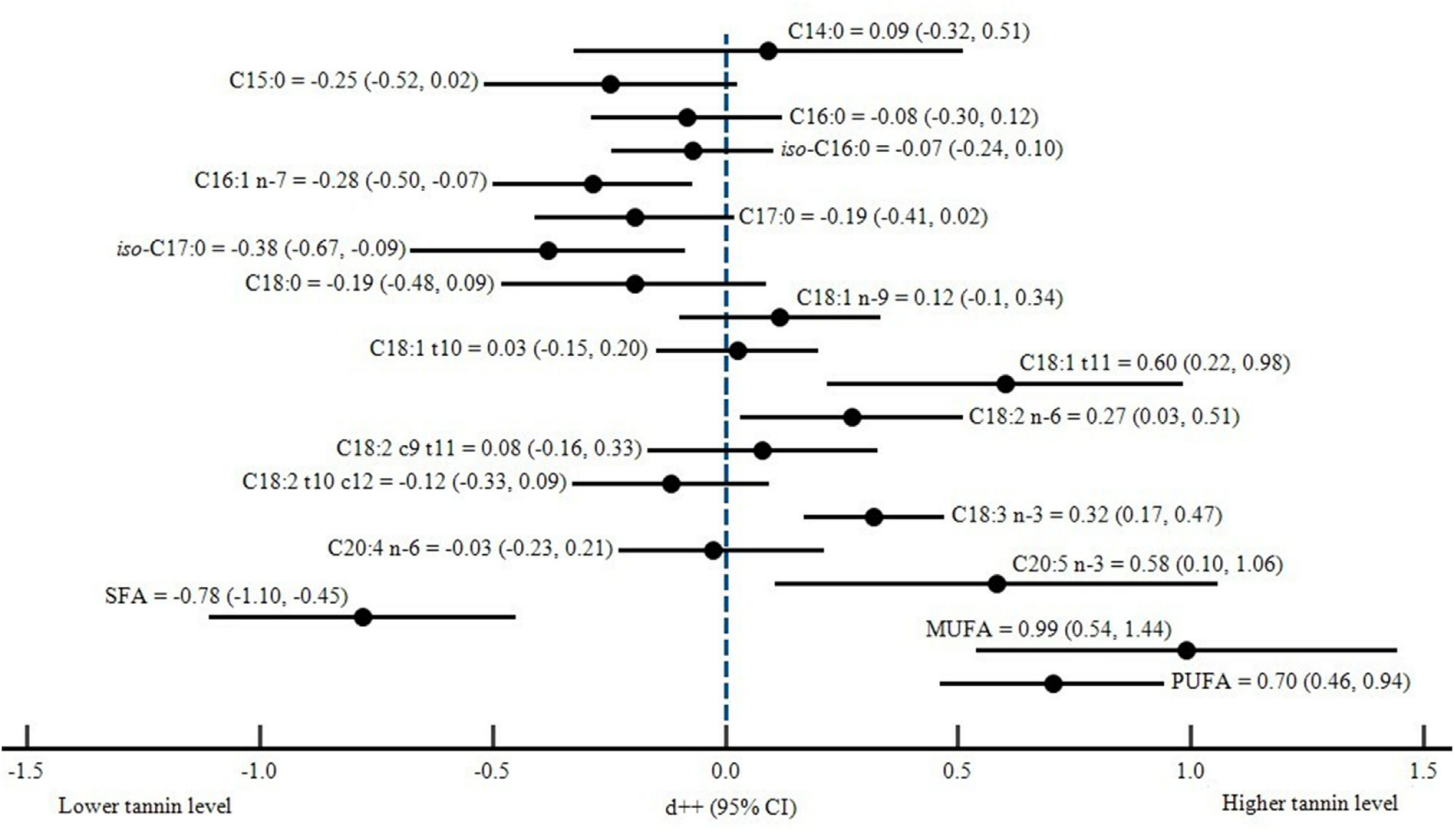
Cumulative forest plot for effect size of the rumen fatty acid profile.

Dietary tannins fell (p < 0.05) C16:1 n-7 and *iso*-C17:0 (Table 4), while the presence of tannins increased (p = 0.002) the intermediate fatty acid of rumen BH, i.e., C18:1 t11. There was also an increase in the PUFA group, i.e., C18:3 n-3 and C20:5 n-3 (p < 0.001 and p = 0.016, respectively) due to tannins, while C18:1 n-9 and C18:2 n-6 were similar in the control and tannin groups. Furthermore, tannins in diets significantly reduced (p < 0.001) SFA composition and simultaneously increased (p < 0.001) MUFA and PUFA in the rumen. An illustration of the cumulative forest plots of the effect size of various types of rumen fatty acids is shown in Figure 3.

**Table 4:** Effect of dietary tannin on the *in vitro* rumen fatty acid profile and BH C18 UFA.

Variable	NC	Estimate	Lower bound	Upper bound	Std. error	*p*-Value	τ^2^	Q	Het. *p*-value	I^2^
Rumen FA profile (% total FA)										
C14:0	16	0.093	−0.325	0.511	0.213	0.662	0.450	59.325	<0.001	74.716
C15:0	56	−0.247	−0.519	0.025	0.139	0.075	0.820	359.701	<0.001	84.710
C16:0	16	−0.082	−0.288	0.124	0.105	0.434	0.022	17.235	0.305	12.966
*iso*-C16:0	34	−0.069	−0.243	0.105	0.089	0.437	0.034	38.493	0.235	14.269
C16:1 n-7	32	−0.284	−0.499	−0.070	0.109	0.009	0.000	20.015	0.935	0.000
C17:0	27	−0.193	−0.408	0.021	0.109	0.078	0.032	29.279	0.299	11.198
*iso*-C17:0	32	−0.381	−0.676	−0.087	0.150	0.011	0.390	85.719	<0.001	63.835
C18:0	58	−0.195	−0.479	0.089	0.145	0.178	0.814	233.476	<0.001	75.584
C18:1 n-9	64	0.117	−0.099	0.334	0.110	0.287	0.265	108.632	<0.001	43.847
C18:1 t10	16	0.027	−0.147	0.200	0.088	0.763	0.000	13.015	0.601	0.000
C18:1 t11	30	0.601	0.218	0.984	0.195	0.002	0.824	151.394	<0.001	80.845
C18:2 n-6	25	0.271	0.033	0.510	0.122	0.026	0.132	41.561	0.014	42.253
C18:2 c9 t11	76	0.080	−0.166	0.327	0.126	0.523	0.765	414.743	<0.001	81.917
C18:2 t10 c12	59	−0.117	−0.329	0.095	0.108	0.278	0.289	110.528	<0.001	47.525
C18:3 n-3	70	0.319	0.168	0.470	0.077	<0.001	0.209	179.237	<0.001	61.503
C20:4 n-6	25	−0.026	−0.230	0.214	0.178	0.806	0.025	26.676	0.320	10.033
C20:5 n-3	21	0.584	0.108	1.060	0.243	0.016	0.846	72.267	<0.001	72.325
SFA	44	−0.778	−1.109	−0.448	0.169	<0.001	0.786	164.466	<0.001	73.855
MUFA	36	0.991	0.538	1.444	0.231	<0.001	1.532	251.871	<0.001	86.104
PUFA	69	0.703	0.461	0.945	0.124	<0.001	0.493	156.843	<0.001	56.645
C18 UFA biohydrogenation (%)										
C18:1 n-9	46	−0.549	−0.957	−0.141	0.208	0.008	1.359	221.482	<0.001	79.682
C18:2 n-6	53	−0.621	−1.051	−0.191	0.219	0.005	1.898	398.590	<0.001	86.954
C18:3 n-3	61	−0.693	−1.097	−0.289	0.206	<0.001	1.786	585.457	<0.001	89.581

### 3.3 Rumen biohydrogenation of PUFA

The BH levels of C18:1 n-9, C18:2 n-6, and C18:3 n-3 in the tannin treatments were 42.38, 64.56, and 70.04%, respectively (Table 2). Meanwhile, the results of the meta-analysis in Table 4 show that dietary tannins inhibited the rumen BH activity of PUFA, as indicated by the lower C18:1 n-9 (p = 0.008), C18:2 n-6 (p = 0.005), and C18:3 n-3 (p < 0.001) in comparison to the control group.

### 3.4 Meta-regression

**Figure 4:**
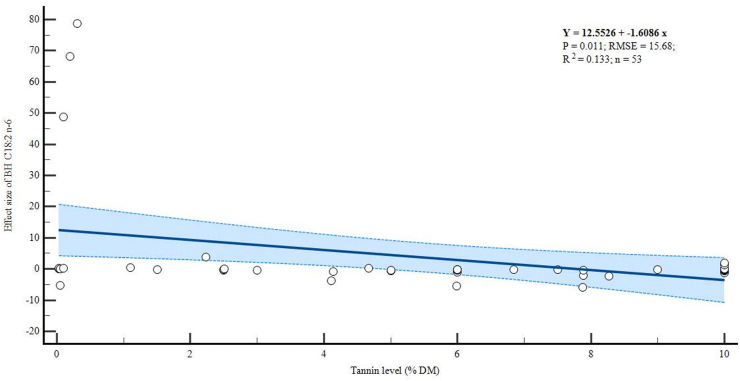
Meta-regression plot of *in vitro* biohydrogenation (BH) C18:2 n-6.

**Figure 5:**
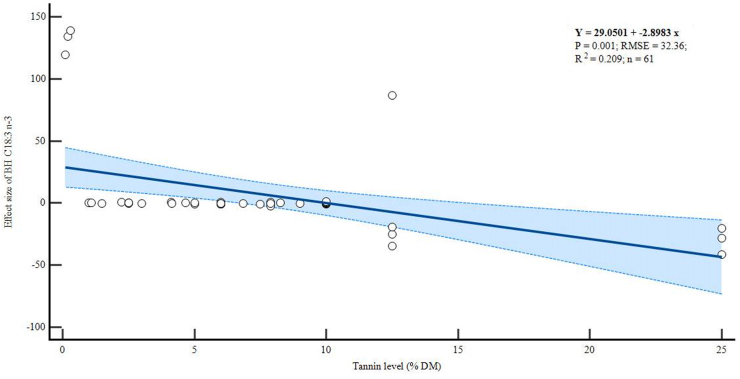
Meta-regression plot of *in vitro* biohydrogenation (BH) C18:3 n-3.

**Figure 6:**
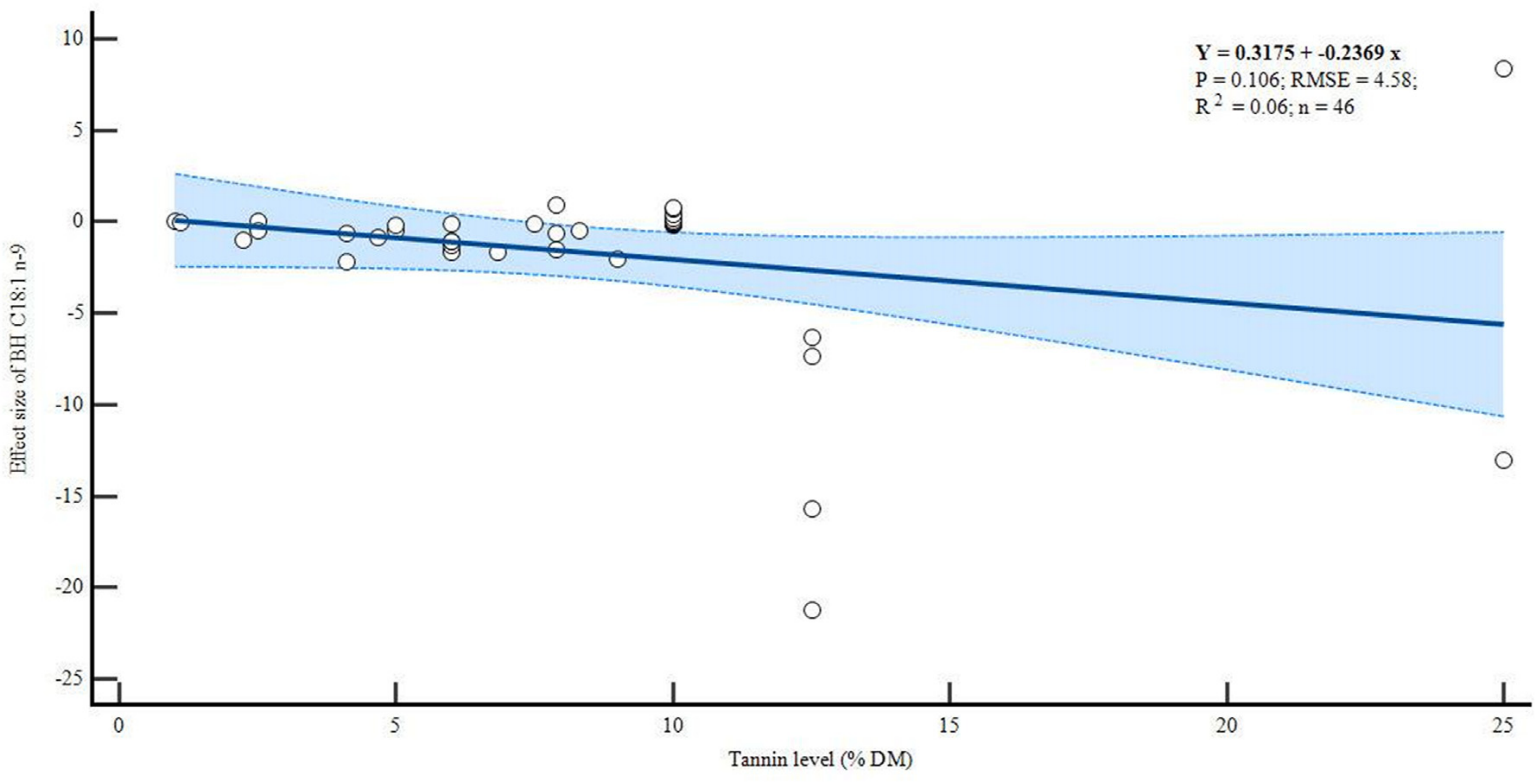
Meta-regression plot of *in vitro* biohydrogenation (BH) C18:1 n-9.

There were negative linear relationships between dietary tannin levels and the BH of C18:2 n-6 (p = 0.011, R^2^ = 0.133) and C18:3 n-3 (p = 0.001, R^2^ = 0.209) as shown in Figures 4 and 5 respectively. However, increasing levels of tannins did not alter the BH of C18:1 n-9 (p = 0.106, R^2^ = 0.06), although the trend was also negative (Figure 6).

### 3.5 Evaluation of publication bias

**Figure 7:**
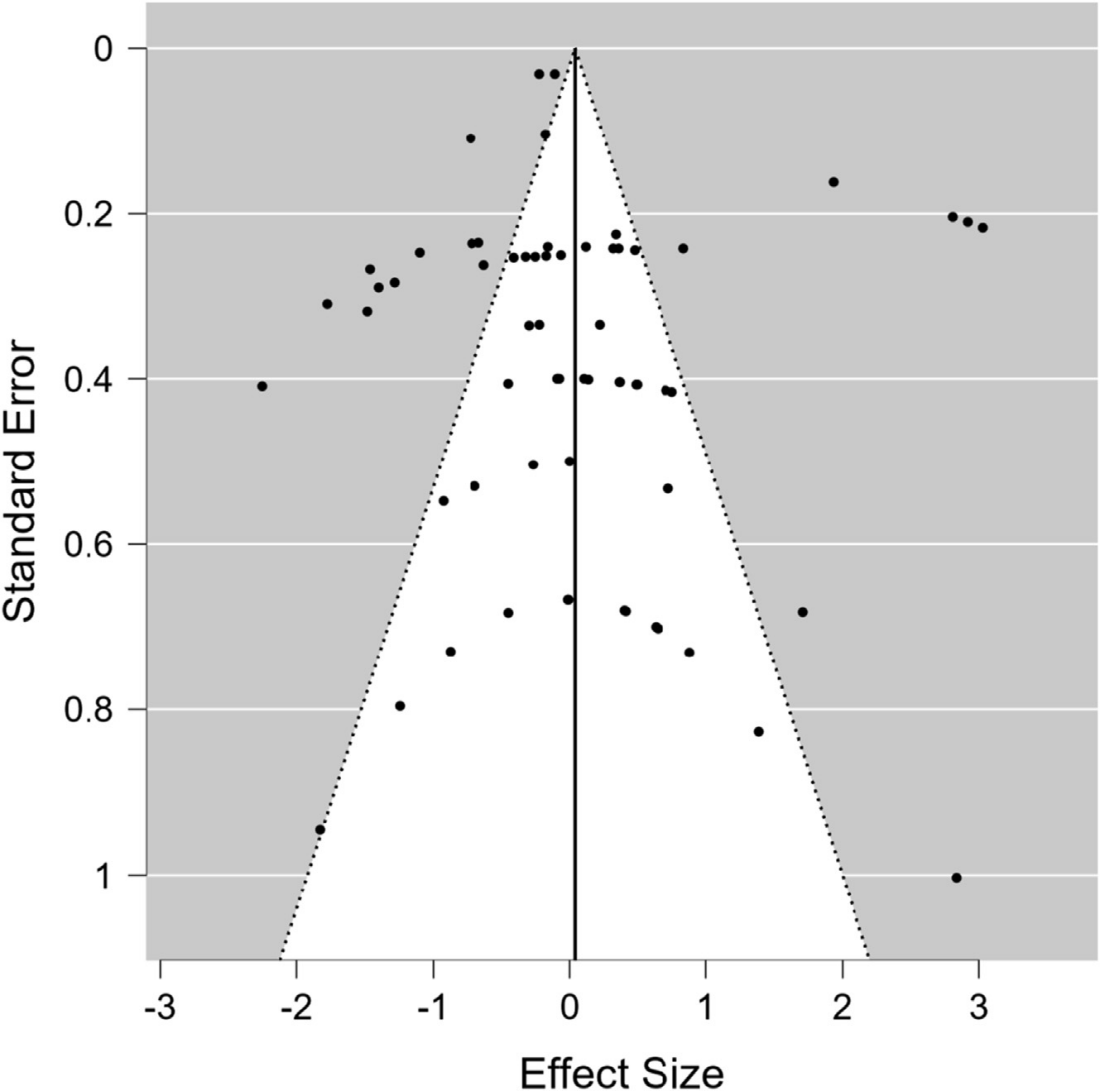
Funnel plot for total volatile fatty acid.

The funnel plot of total VFA, which is the main parameter in rumen fermentation, showed symmetrical values (Figure 7). This was supported by a statistical assessment of publication bias using Egger's test, which showed a non-significant result (p = 0.905), indicating that no publication bias existed in the meta-analysis study.

## 4 Discussion

The meta-analysis results on the rumen fermentation profile obtained in this study are relatively similar to those of Jayanegara et al. [43]. They show a relationship between an increase in tannin levels with decreased production levels of methane, ammonia, total bacteria, and *iso*-SCFA, but not in relation to pH parameters *in vitro*. This was due to the protective effect of the tannin component through the production of stable formations with easily degraded proteins in the rumen [44]. The effect of tannins in the rumen environment also caused abnormalities in the morphology and cell growth of some microbes, which led to a modulation of fermentation [45]. The bioactivity of tannin components such as gallotannins and ellagitannins was able to inhibit the growth of broad-spectrum bacterial species through the disruption of cell regulatory mechanisms [46]. This is in line with a study by Mazhangara et al. [47], which showed the gram-negative and positive antibacterial potential from a crude extract of *Teucrium trifidum* (CT 77.34–99.40 mg CE/g).

The effect of condensed tannin (CT) on the rumen showed a significant reduction in the accumulation of rumen ammonia compared to its effect on protozoa depopulation [48]. According to Zhou et al. [49], the supplementation level of 16.9 tannic acid/kg DM in combination with dietary protein produced an inhibitory effect on fermentation activity and rumen degradation, which did not affect the population of the genus *Butyrivibrio*. Tannin treatments also showed a consistent trend of population decline for rumen microbes associated with the BH process [50]. Few studies have been conducted on the tannin mechanism in inhibiting *B*. *fibrisolvens* related to rumen BH. Early indications show that tannin changes the pattern of hydrogen ion production, disintegrates cell walls and liposome, disrupts oxidative phosphorylation metabolic pathways, and reduces substrates for bacterial growth directly related to the rumen FA metabolism [51, 52]. On the other hand, the molecular weight, dose, and type of tannin supplemented in the diet are amongst factors that determine the effectiveness of tannin in influencing the rumen microbial community selectively. This indicates that there are variations in some rumen fermentation profile variables in certain tannin level categories between the *in vitro* and *in vivo* rumen studies. The study by Arcuri et al. [53] found that the *B*. *fibrisolvens* and *Streptococcus bovis* strains isolated from Holstein × Zebu cattle resisted the biological effect of CT *Mimosa artemisia* extract at low levels (2–3 g L^−1^). The effect of eliminating the rumen methanogen population using tannin-sourced feeds has a direct impact on reducing the overall level of ruminant methane production. This is because approximately 37% of rumen methane emissions come from an active community of methanogenic bacteria that are in symbiosis with rumen protozoa [54]. In addition, a meta-analysis study of 30 articles on the estimation of rumen methane gas production with different systems showed that the *in vitro* system experienced measurement bias at the level of tannin addition of above 100 g tannin/kg DM [55]. Therefore, *in vitro* research is a more appropriate choice to initiate an exploratory study on the potential of feed ingredients in ruminants.

Studies on the effect of bioactive plant components such as tannins are among the promising strategies for avoiding rumen BH of beneficial fatty acid groups. It has also been shown that different sources of tannins are effective in suppressing BH and C18:0 production [19], where various tropical and subtropical plant species have been used effectively [6, 55]. This shows that tannins have an impact on increasing the composition of C18 UFA and C18:1 t11. Furthermore, the mechanism of tannins in suppressing the BH process can take place through the inhibition of the lipolysis process, which is the initial stage of the decomposition of fat fraction into free fatty acids. There is also a toxic effect of tannins on rumen microbes in the terminal stage of BH, which has a positive impact on increasing the percentage of rumen intermediate fatty acids [56]. Similarly, a meta-analysis of 12 articles on natural rumen biomodifiers, for example chitosan, showed similar characteristics to tannin supplementation, such as reduced SFA, and increased rumen CLA and PUFA with *in vitro* batch culture [57].

Based on the consistency of the findings of previous studies, diets rich in polyphenols have inhibitory effects on rumen BH and methanogenesis [8]. A meta-analysis study of 38 articles showed that increasing the levels of tannin supplementation (0.1–20 g/kg DM) also optimised the accumulation of conjugated linoleic acid (CLA) in *in vitro* and *in vivo* observations [58]. The study showed variations in measuring the true production of rumen fatty acids in different observational models. This is a limitation of our study, which only focused on *in vitro*. Enjalbert et al. [59] found differences in the rates of *in vitro* and *in situ* rumen BH and concluded that both methods could still feasibly used. This was further emphasized by Fievez et al. [60], who measured the rumen BH activity of unprotected fatty acid sources using several approaches. They found that the BH profile of C18 UFA and C18:0 production from simulated continuous cultures for 24 h showed values approximating to those observed *in vivo*.

Furthermore, *Carica papaya* leaf supplementation with CT levels of 17.39 mg/g DM to BH levels of C18:2 n-6 and C18:3 n-3 in different rumen systems showed the same significance level [10, 27]. However, these results were not in line with the rumen CLA production level at various incubation periods. Meanwhile, a clearer negative correlation based on the increase in dietary tannin levels in the BH activity of C18 UFA is the accumulation of various tannin effects on the metabolic pathways of rumen fatty acids. The difference in the degree of saturation between unsaturated fatty acids also contributes to the extensive BH. For comparison in the plant secondary metabolite, the effect of adding triterpene saponin extract (500–1000 mg/l) on *in vitro* BH of C18:2 n-6 was 76.6–78.1%, while C18:3 n-3 was 85.3–86.6% [61]. According to Eburu and Anya [62], the addition of 300 mg/l essential oil such as anise, lavender, and mixed anise-lavender showed BH levels *in vitro* (24 h) of C18:2 n-6 of 40–68.1 % and C18:3 n-3 of 42.1–70.2%. These findings at least show that the effectiveness of tannins in suppressing rumen BH activity is between saponins and essential oils.

## 5 Conclusion

This study has revealed that dietary tannins enhance the accumulation of C18:1 t11, C18:3 n-3, C20:5 n-3, MUFA, and PUFA proportions in the rumen. Based on rumen BH activity, an increase in tannin levels linearly reduces the BH of C18:2 n-6 and C18:3 n-3. These patterns suggest that tannins may elevate the PUFA and lower SFA contents in animal products, which, in turn, may improve human health. In addition, tannins provide beneficial effects in relation to a number of rumen fermentation and microbial population parameters, as indicated by reduced ammonia concentration, methane emissions, protozoa population, methanogens, and *B*. *fibrisolvens* bacteria. The ability of tannins to lower methane emissions would further help the mitigation of greenhouse gas emissions from the livestock sector and their accumulation in the atmosphere. Further investigation is required to identify tannin types and levels that effectively alter rumen lipid metabolism using a research synthesis approach.

## Declarations

### Author contribution statement

Malik Makmur: Performed the study; Analyzed and interpreted the data; Wrote the paper.

Mardiati Zain: Contributed analysis tools or data; Wrote the paper.

Muhammad Miftakhus Sholikin: Performed the study; Analyzed and interpreted the data; Wrote the paper.

Suharlina: Performed the study; Contributed analysis tools or data; Wrote the paper.

Anuraga Jayanegara: Conceived and designed the study; Analyzed and interpreted the data; Wrote the paper.

### Funding statement

The first author is grateful to IPB University, Indonesia, for providing a post doctoral grant in 2021 with contract number 2/IT3/WCU/2021. All authors are grateful to the Ministry of Education, Culture, Research and Technology, Republic of Indonesia, for funding the study through “Penelitian Dasar Kompetitif Nasional” scheme, year 2022, contract number 082/E5/PG.02.00.PT/2022.

### Data availability statement

Data will be made available on request.

### Declaration of interests statement

The authors declare no conflict of interest.

### Additional information

Supplementary content related to this article has been published online at https://doi.org/10.1016/j.heliyon.2022.e09828.
