# Supplementary material for: Modulatory effects of dietary tannins on polyunsaturated fatty acid biohydrogenation in the rumen: A meta-analysis
Source: Heliyon. 2022 Jun 29;8(7):e09828. doi: 10.1016/j.heliyon.2022.e09828 (PMC9263859; doi:10.1016/j.heliyon.2022.e09828)
Supplement: Multimedia component 1 [file mmc1.pdf]

No. 221143-BPS-331

Britannia
Proofreading Service

Certificate of English Editing

This is to certify that the manuscript:

Modulatory effects of dietary tannins on polyunsaturated fatty acid biohydrogenation in the rumen: A meta-analysis

By the author(s):

Malik Makmur, Mardiati Zain, Muhammad Miftakhus Sholikin, Suharlina, Anuraga Jayanegara

has been edited for English language usage
by a native British proofreader of Britannia Proofreading Service.

Ilham Reza Ferdian
Director

Date: 22 June 2022

www.britanniaproofreadingservice.com

Disclaimer: The author is free to accept or reject our changes in the document after our editing. However, we do not bear responsibility for revisions made to the document after our edit on 18 June 2022.
